# Occurrence of *Pasteurella multocida* among pigs with respiratory disease in China between 2011 and 2015

**DOI:** 10.1186/s13620-016-0080-7

**Published:** 2017-01-10

**Authors:** Huisheng Liu, Zhanqin Zhao, Xiaojian Xi, Qiao Xue, Ta Long, Yun Xue

**Affiliations:** 1 Laboratory of Veterinary Microbiology, College of Animal Science and Technology, Henan University of Science and Technology, Luoyang, China; 2 Laboratory of Medical Engineering, College of Medical Technology and Engineering, Henan University of Science and Technology, Luoyang, China

**Keywords:** *Pasteurella multocida*, *Bordetella bronchiseptica*, Swine, Respiratory pathogen, Co-infection

## Abstract

**Background:**

Prior to the 1990s, *P. multocida* capsular serogroup A was the most prevalent in China, followed by serogroups B and D. Thirty years later, serogroup D became the most prevalent, followed by serogroups A and B. However, the *P. multocida* capsular serogroups currently circulating in China remain unclear. Therefore, the aim of the present study was to provide an update on *P. multocida* serogroups isolated from diagnostic samples collected from clinically diseased pigs in Central and Eastern China from 2011 to 2015.

**Results:**

Between February 2011 and October 2015, 296 isolates of *Pasteurella multocida* were collected from 3212 pigs with clinical respiratory disease in 12 provinces of China (isolation rate of 9.2%). Of the 296 collected isolates, 146 (49.3%) were *P. multocida* capsular type A, 141 (47.6%) were capsular type D, and one was capsular type B. *Streptococcus suis* (94/193; 48.7%), *Haemophilus parasuis* (76/193; 39.3%), *Escherichia coli* (53/193; 27.5%), and *Bordetella bronchiseptica* (26/193; 13.5%) were frequently isolated together with *P. multocida.* A total of 14 toxigenic *P. multocida* strains co-isolated with other pathogens from 32 cases of atrophic rhinitis were classified into serogroup D. The virulence of *P. multocida* capsular type A isolates was higher than that of capsular type D isolates based on LD_50_ studies in mice.

**Conclusions:**

Over the past 5 years, *P. multocida* capsular type A was the most frequently isolated from diagnostic submissions in Central and Eastern China, followed by serogroups D and B.

## Background

*Pasteurella multocida* (*P. multocida*), a Gram-negative, non-motile, facultative coccobacillus, belonging to the *Pasteurellaceae* family [1], is an opportunistic pathogen and common inhabitant of the upper respiratory tract of many animal species, and a causative agent of numerous economically important diseases worldwide, including atrophic rhinitis (AR) in swine, fowl cholera in birds, pneumonia and shipping fever in cattle, snuffles in rabbits [2–4], and occasional zoonotic infections in humans [5].

*P. multocida* strains are classified into five capsular serogroups (A, B, D, E, and F) and 16 somatic serotypes (1 to 16) based on lipopolysaccharide antigens [6–8]. To date, capsular serogroups A, B, and D have been identified in pigs [8, 9]. Of these, serogroups A and D are causative agents of AR, which result in significant economic losses to the swine industry worldwide [9–12].

*P. multocida* possesses various virulence factors, including fimbriae, adhesins, and toxins, especially dermonecrotic toxin. Of these virulence factors, toxins produced by serogroups A and D play important roles in the pathogenicity of *P. multocida*. Only toxigenic strains can cause AR, which is characterized by loss of the nasal turbinate bone [13, 14]. Therefore, data on *P. multocida* toxins can provide better understanding of pasteurellosis in pigs.

Diseases caused by different serogroups and their prevalence may change over time within particular regions. Prior to the 1990s, capsular serogroups A and B were the most prevalent in diseased pigs in China, followed by serogroup D [15]. Thirty years later, serogroups A and D became the most prevalent, followed by serogroup B [8]. However, these data are from several years ago and do not represent *P. multocida* capsular serogroups circulating in China in recent years, as the identification of *P. multocida* capsular serogroups currently circulating in China remain unclear. Hence, to provide an update on *P. multocida* capsular serogroups currently circulating in China, we investigated a total of 296 *P. multocida* strains isolated from pigs submitted to our diagnostic laboratory from 12 provinces of China from 2011 to 2015 to estimate the occurrence of *P. multocida* alone and in combination with other bacterial pathogens in pigs with respiratory disease. The findings of this study provide useful data to inform control strategies for respiratory disease and are relevant to future work on the development of effective vaccines.

## Methods

### Samples collection

Between February 2011 and October 2015, a total of 3212 lung samples were collected from pigs submitted to our diagnostic laboratory from 12 different provinces in Central and Eastern China for further identification of *P. multocida*. The data obtained from clinically affected pigs in Central and Eastern China may reflect, at least in part, the true occurrence of *P. multocida* in diagnostic submissions from the whole of China, since most of the pigs were raised in Central and Eastern China. All lung samples exhibited different degrees of pneumonic lesions and were collected by a single skilled veterinarian.

A total of 713 commercial pig farms were included, which had a wide variety of management types and herd size with small-, medium- and large-scale pig farms as well as various types of backyard farms. Only one pig farm was selected per region. If there was more than one pig farm in the same region, the larger pig farm was selected. During the study period, 13 to 107 pig cases were collected per month. One to 10 lung samples were randomly collected per herd. If more than one *P. multocida* isolate was found in the same herd, one of the isolates belonging the same serogroup was included in the analysis. All samples were processed for bacterial culture within 10 h of collection.

### Bacterial isolation and identification

Each sample was plated on tryptic soy agar supplemented with 10 μg/mL of nicotinamide adenine dinucleotide and 5% fetal calf serum. All plates were incubated at 37 °C for 24–48 h. Afterward, the isolates were purified and cultured by standard methods for rapid primary identification of *P. multocida*, *Streptococcus suis* (*S. suis*), *Haemophilus parasuis* (*HPS*), *Escherichia coli* (*E. coli*), *Bordetella bronchiseptica* (*Bb*), *and Salmonella* spp., respectively, by polymerase chain reaction (PCR) using the respective specific primers listed in Table 1, as described previously [16–21]. After this stage, the strains were further identified by Gram-staining characteristics and oxidase (Gram-negative bacilli) or catalase tests [22]. All primers were synthesized by Sangon Biotech Co., Ltd. (Shanghai, China). Standard *P. multocida*, *E. coli*, and *Salmonella* strains, purchased from China Institute of Veterinary Drug Control (Beijing, China), and *Bb* and *HPS* strains, stored at our laboratory [23], were used as positive controls. Sterile water was used as a negative control. All isolates were freeze-dried and stored at –80 °C.

**Table 1:** Primers used for the identification of bacterial isolates, capsule typing, and the *toxA* gene

Strains	Target genes	Name	Sequences (5’ → 3’)	Product (bp)	References
*P. multocida*	kmt1	Pm-1	ATCCGCTATTTACCCAGTGG	457	[17]
		Pm-2	GCTGTAAACGAACTCGCCAC		
*P. multocida* (serotype A)	hyaD-hyaC	APm-1	GATGCCAAAATCGCAGTCAG	1048	[7]
		APm-2	TGTTGCCATCATTGTCAGTG		
*P. multocida* (serotype B)	dcbD	BPm-1	CATTTATCCAAGCTCCACC	758	[7]
		BPm-2	GCCCGAGAGTTTCAATCC		
*P. multocida* (serotype D)	dcbF	DPm-1	TTACAAAAGAAAGACTAGGAGCCC	647	[7]
		DPm-2	CATCTACCCACTCAACCATATCAG		
*P. multocida* (serotype E)	*ecbJ*	EPm-1	TCCGCAGAAAATTATTGACTC	512	[7]
		EPm-2	GCTTGCTGCTTGATTTTGTC		
*P. multocida* (serotype F)	*fcbD*	FPm-1	AATCGGAGAACGCAGAAATCAG	852	[7]
		FPm-2	TTCCGCCGTCAATTACTCTG		
*P. multocida* (*toxA* gene)	toxA	toxA-1	CTTAGATGAGCGACAAGG	864	[24]
		toxA-2	GAATGCCACACCTCTATAG		
*S. suis*	gdh	Ss-1	GCAGCGTATTCTGTCAAACG	689	[20]
		Ss-2	CCATGGACAGATAAAGATGG		
*HPS*	16S rRNA	HPS-1	TATCGRGAGATGAAAGAC	1086/1090	[21]
		HPS-1’	GTAATGTCTAAGGACTAG		
		HPS-2	CCTCGCGGCTTCGTC		
*E. coli*	uidA	Ec-1	ATGAAAGCTGGCTACAGGAAGGCC	264	[16]
		Ec-2	GGTTTATGCAGCAACGAGACGTCA		
*Bb*	flaB	Bb-1	TGGCGCCTGCCCTATC	237	[19]
		Bb-2	AGGCTCCCAAGAGAGAAA		
*Salmonella*	invA	Sal-1	CAGGATACCTATAGTGCTGC	580	[18]
		Sal-2	CGCACCGTCAAAGGAACCGT		

*S. suis*, *HPS*, *E. coli*, and *Bb* represent *Streptococcus suis*, *Haemophilus parasuis*, *Escherichia coli* and *Bordetella bronchiseptica*, respectively

### Capsule typing

The capsular types of *P. multocida* were confirmed by multiplex capsule PCR with capsule-specific primer pairs (Table 1), as described by Townsend et al. [7].

### Detection of *toxA* gene

The *toxA* gene of *P. multocida* was confirmed by PCR with a specific primer pair (Table 1), as described by Zhao et al. [24].

### Detection of virulence

Twenty-five BALB/c mice at 6 weeks of age (Vital River Laboratories Co., Ltd., Beijing, China) were randomly assigned to five groups (A1–A5) of five animals each, as previously described [25]. The mice were housed in a standard animal facility with *ad libitum* access to normal rodent diet and water. *P. multocida* was cultured on trypticase soy broth supplemented with 5% fetal calf serum and incubated on a shaking table at 200 rpm and temperature of 37 °C for 12–16 h. The number of *P. multocida* were enumerated by plate counts. Cultures were serially diluted 5-fold in sterile phosphate buffered saline (PBS). Then, the original cultures (undiluted), cultures diluted 5-fold, cultures diluted 25-fold, and cultures diluted 125-fold were generated. The mice in groups A1–A4 were inoculated intraperitoneally with 0.2 ml per mouse of the original cultures, and cultures diluted 5, 25, and 125-fold, respectively, and the mice in group A5 were injected with 0.2 ml of sterile PBS, as a control group. The 50% lethal dose (LD_50_) was calculated on 15 days post-infection, as previously described [25].

### Statistical analysis

Statistical analysis was performed using SPSS version 17.0 software (SPSS Inc., Chicago, IL, USA). A *p*-value <0.05 was considered statistically significant.

## Results

### Prevalence of *P. multocida* in porcine clinical samples

**Fig. 1:**
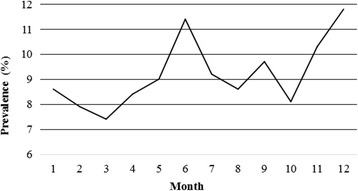
Distribution of *P. multocida* isolates by the sampling month between February 2011 and October 2015. The isolation rates were 19/222, 20/253, 22/296, 25/297, 21/233, 38/332, 28/303, 24/280, 21/217, 21/260, 29/281, and 28/238 for months 1–12, respectively. The isolation rates given are the average isolation rates per month over the 5-years study period. The highest isolation rates were recorded in month 12, followed by months 6, 11, and 9. However, as a whole, differences in the isolation rates were not significant (*p* > 0.05). Statistical analysis was performed using the *χ* ^2^ test

Over the 5-year period of this study, *P. multocida* was isolated from 296/3212 (9.2%) lung samples collected from pigs with clinical respiratory disease. The annual isolation rates ranged from 7.5% to 10.7% with the highest isolation rate recorded in 2012 and the lowest in 2011 (Table 2). Differences in the isolation rates were not significant (*p* > 0.05) between February 2011 and October 2015. There was no association between collection month and isolation rate (*p* > 0.05). The monthly isolation rates ranged from 7.4% to 11.8% with the highest recorded in month 12, followed by months 6, 11, and 9 (Fig. 1). In addition, strains of *P. multocida* were isolated throughout the year without seasonal variation (*p* > 0.05; spring, months 3–5; summer, months 6–8; autumn, months 9–11; winter, months 12–2; Fig. 1).

**Table 2:** Numbers of isolates of *P. multocida* and other pathogens detected in 3212 lung samples from pigs collected between February 2011 and October 2015

Year	No. of sample	No. of *P. multocida*	Isolation rates(%)^a^	Pathogens co-infected with *P. multocida*					
				*S.suis*	*HPS*	*E. coli*	*Bb*	*Salmonella*	Others
2011	616	46	7.5	15	13	8	3	2	2
2012	570	61	10.7	20	17	11	4	2	4
2013	630	57	9.0	19	15	11	6	1	3
2014	754	72	9.6	22	17	12	7	3	6
2015	642	60	9.3	18	14	11	6	2	4
Total	3212	296	9.2	94	76	53	26	10	19
				(48.7%)	(39.3%)	(27.5%)	(13.5%)	(5.1%)	(9.8%)

^a^There were no significant differences in the annual isolates rates of *P. multocida* between 2011 and 2015 (*p* > 0.05). Statistical analysis was performed using the *χ* ^2^ test. *S. suis*, *HPS*, *E. coli*, and *Bb* represent *Streptococcus suis*, *Haemophilus parasuis*, *Escherichia coli* and *Bordetella bronchiseptica*, respectively

*P. multocida* strains of capsular type A were isolated from 146 lung samples (49.3%), capsular type D strains were isolated from 141 lung samples (47.6%), capsular type B strains were isolated from one lung sample, and eight isolates were untypeable. Capsular types E and F were not detected in this study.

### Prevalence of *P. multocida* accompanied by other species

*P. multocida* was isolated together with other bacterial pathogens in 65.2% (193/296) of the lung samples (Table 2). *S. suis* (94; 48.7%), *HPS* (76; 39.3%), and *E. coli* (53; 27.5%) were most frequently isolated together with *P. multocida,* followed by *Bb* (26; 13.5%) and *Salmonella* spp. (10; 5.1%). *S. suis* was isolated from 25.3% (812/3212) of the lung samples, *HPS* was isolated from 16.6% (533/3212), *E. coli* was isolated from 12.9% (415/3212), *Bb* was isolated from 7.3% (236/3212), and *Salmonella* was isolated from 3.3% (107/3212). *P. multocida* was the fourth most frequently isolated bacteria after *S. suis*, *HPS* and *E. coli*.

### Prevalence of toxigenic *P. multocida* accompanied by other species

Of the 296 *P. multocida* isolates, only 14 toxigenic strains of *P. multocida* were identified by PCR and all were classified as capsular type D. Over the 5-year period of this study, 32/3212 (1%) pigs with typical clinical signs of AR were identified. All toxigenic strains of *P. multocida* were isolated from pigs with clinical signs of AR. Ten of these toxigenic *P*. multocida strains were isolated with *Bb*, seven with *HPS*, three with *S. suis*, and one with *E. coli*. In addition, *Bb* was isolated from pigs with no infection of toxigenic *P. multocida* strains.

### Virulence

Twenty strains of *P. multocida* of each capsular types A and D were randomly selected, respectively, to estimate the LD_50_ in mice. Twenty-five mice were used for each of these 40 isolates (1000 mice in total). The results showed that the virulence of only one isolate of *P. multocida* capsular type A was relatively weak with a LD_50_ of 2.1 × 10^5^, and the virulence of PM-30 of *P. multocida* capsular type D strain was lowest with a LD_50_ of > 4.9 × 10^7^. As a whole, the virulence of *P. multocida* capsular type A was higher than that of capsular type D (Table 3).

**Table 3:** Virulence of *P. multocida* capsular type A and D strains in mice

Strain	Capsular type	Origin	LD_50_ (CFU)	Strain	Capsular type	Origin	LD_50_ (CFU)
PM-1	A	Henan	<84	PM-21	D	Henan	1.3 × 10^6^
PM-2	A	Shanxi	<23	PM-22	D	Hubei	3.4 × 10^6^
PM-3	A	Hubei	4.1 × 10^3^	PM-23	D	Henan	1.9 × 10^5^
PM-4	A	Henan	<16	PM-24	D	Sichuan	2.1 × 10^5^
PM-5	A	Shanxi	<38	PM-25	D	Hubei	2.6 × 10^4^
PM-6	A	Anhui	<167	PM-26	D	Shanxi	2.6 × 10^4^
PM-7	A	Henan	<68	PM-27	D	Henan	5.7 × 10^5^
PM-8	A	Sichuan	<145	PM-28	D	Henan	2.2 × 10^6^
PM-9	A	Shanxi	<17	PM-29	D	Hubei	3.3 × 10^6^
PM-10	A	Anhui	104	PM-30	D	Henan	>4.9 × 10^7^
PM-11	A	Henan	7.2 × 10^4^	PM-31	D	Shandong	3.3 × 10^6^
PM-12	A	Shanxi	<69	PM-32	D	Henan	1.6 × 10^7^
PM-13	A	Henan	6.8 × 10^4^	PM-33	D	Sichuan	5.5 × 10^5^
PM-14	A	Shanxi	<103	PM-34	D	Hubei	2.9 × 10^5^
PM-15	A	Henan	123	PM-35	D	Henan	1.2 × 10^7^
PM-16	A	Hubei	<134	PM-36	D	Shanxi	1.3 × 10^5^
PM-17	A	Shanxi	<70	PM-37	D	Henan	1.6 × 10^5^
PM-18	A	Henan	<42	PM-38	D	Shandong	5.3 × 10^5^
PM-19	A	Henan	2.0 × 10^4^	PM-39	D	Henan	4.4 × 10^4^
PM-20	A	Hubei	<93	PM-40	D	Jiangsu	5.4 × 10^5^

PM represent *Pasteurella multocida*

## Discussion

In China, as in other parts of the world, *P. multocida* is frequently associated with outbreaks of respiratory infections in pigs. In this study, the prevalence of *P. multocida* infection was 9.2% (296/3212), which was higher (*p* > 0.05, *χ* ^2^ test) than in previous reports (8.0%, 233/2912) from China [8]. Additionally, the *χ* ^2^ test results showed that the annual prevalence of *P. multocida* infection was relatively stable (*p* > 0.05) between 2011 and 2015, in accordance with the annual prevalence from 2003 to 2007, as reported by Tang et al. [8]. The results of this study showed that the prevalence of *P. multocida* infection did not significantly differ (*p* > 0.05) by season, in agreement with the report by Tang et al. [8]. In addition, there was no clear association of *P. multocida* infection with the sampling month, with the highest prevalence in month 12, followed by month 6. However, because only a few samples fron each month were assayed, the data in this study may not reflect the real differences between months. Hence, the difference in isolation rates between months should to be addressed in future studies.

Our findings suggest that strains of *P. multocida* are widely prevalent on pig farms, and there were no differences in the prevalence (*p* > 0.05, *χ* ^2^ test) of *P. multocida* of capsular type A (49.3%) and D (47.6%), which was different from the results of previous report from China (39.5% vs. 54.9%, *p* < 0.05) [8] and different from the report by Rajkhowa et al. [26]. These results showed that the prevalence of *P. multocida* capsular type A strains exhibited a tendency to increase over the past 5 years in China. The reasons for this tendency were unclear and need to be addressed in future studies. The eight untypeable isolates, which were associated with pneumonic pasteurellosis, should be further investigated.

In this study, *S. suis*, *HPS*, *E. coli*, and *Bb* were frequently isolated together with *P. multocida*. Similar results were reported by Tang et al. [8] and Zhao et al. [22]. Accumulating evidence suggests that mixed infection of two or more bacteria may have been common over the last decade in China. Therefore, the practical significance of this finding is that future work on control of disease caused by *P. multocida* in pigs may require investigation of multivalent vaccines based on these bacteria rather than a monovalent vaccine. In addition, our results showed high isolation rates of *S. suis*, *HPS*, *E. coli*, and *Bb*, in accordance with recent reports from China [8, 22] and some other countries [27–29].

AR is seldom reported in China, which is supported by our findings (32/3212) as well as the reports by Tang et al. [8] and Zhao et al. [22]. The findings of previous studies combined with those of the present study confirmed the presence of AR in Henan, Shandong, Fujian, Hainan, Anhui, and Hubei provinces to date. In this study, all 14 toxigenic *P. multocida* strains were type D, which was in accordance with the findings of previous reports [8, 9, 22, 30]. In addition, 14 toxigenic *P. multocida* strains were isolated together with one, two, or three other pathogens and *Bb* was most frequently isolated with toxigenic *P. multocida*.

Previous reports have mainly focused on the detection of virulence genes of *P. multocida* isolates. This is the first large-scale study of the virulence of *P. multocida* isolates based on LD_50_ studies in mice. Our findings showed that the virulence of *P. multocida* capsular type A isolates was higher than that of capsular type D isolates. The high virulence and prevalence of isolates of *P. multocida* capsular type A is expected to increase economic losses to the swine industry in China.

## Conclusions

Our results disclosed epidemiological information of *P. multocida* infection of pigs over the past 5 years in China. Data from a total of 296 *P. multocida* isolates revealed no significant difference in the prevalence of *P. multocida* capsular types A and D in diagnostic procine lung samples submitted from 12 provinces in Central and Eastern China, and *S. suis*, *HPS*, *E. coli*, and *Bb* are often isolated together with *P. multocida*. In addition, our results showed that the virulence of *P. multocida* capsular type A was greater than that of capsular type D. The findings of this study are expected to facilitate a better understanding of the current status of *P. multocida* infection among pigs in China.

## Abbreviations

AR: Atrophic rhinitis
*Bb*: *Bordetella bronchiseptica*
*E. coli*: *Escherichia coli*
*HPS*: *Haemophilus parasuis*
LD_50_: 50% lethal dose
*P. multocida*: *Pasteurella multocida*
PBS: Phosphate buffered saline
PCR: Polymerase chain reaction
*S. suis*: *Streptococcus suis*
